# Evaluation of a Natural Language Processing Approach to Identify Diagnostic Errors and Analysis of Safety Learning System Case Review Data: Retrospective Cohort Study

**DOI:** 10.2196/50935

**Published:** 2024-08-26

**Authors:** Azade Tabaie, Alberta Tran, Tony Calabria, Sonita S Bennett, Arianna Milicia, William Weintraub, William James Gallagher, John Yosaitis, Laura C Schubel, Mary A Hill, Kelly Michelle Smith, Kristen Miller

**Affiliations:** 1 Center for Biostatistics, Informatics, and Data Science MedStar Health Research Institute Washington, DC United States; 2 Department of Emergency Medicine Georgetown University School of Medicine Washington, DC United States; 3 Department of Quality and Safety MedStar Health Research Institute Washington, DC United States; 4 National Center for Human Factors in Healthcare MedStar Health Research Institute Washington, DC United States; 5 Population Health MedStar Health Research Institute Washington, DC United States; 6 Georgetown University School of Medicine Washington, DC United States; 7 Family Medicine Residency Program MedStar Health Georgetown-Washington Hospital Center Washington, DC United States; 8 MedStar Simulation Training & Education Lab (SiTEL) MedStar Institute for Innovation Washington, DC United States; 9 Institute of Health Policy, Management & Evaluation University of Toronto Toronto, ON Canada; 10 Michael Garron Hospital Toronto, ON Canada

**Keywords:** diagnostic error, electronic health records, machine learning, natural language processing, NLP, mortality, hospital, risk, length of stay, patient harm, diagnostic, EHR

## Abstract

**Background:**

Diagnostic errors are an underappreciated cause of preventable mortality in hospitals and pose a risk for severe patient harm and increase hospital length of stay.

**Objective:**

This study aims to explore the potential of machine learning and natural language processing techniques in improving diagnostic safety surveillance. We conducted a rigorous evaluation of the feasibility and potential to use electronic health records clinical notes and existing case review data.

**Methods:**

Safety Learning System case review data from 1 large health system composed of 10 hospitals in the mid-Atlantic region of the United States from February 2016 to September 2021 were analyzed. The case review outcome included opportunities for improvement including diagnostic opportunities for improvement. To supplement case review data, electronic health record clinical notes were extracted and analyzed. A simple logistic regression model along with 3 forms of logistic regression models (ie, Least Absolute Shrinkage and Selection Operator, Ridge, and Elastic Net) with regularization functions was trained on this data to compare classification performances in classifying patients who experienced diagnostic errors during hospitalization. Further, statistical tests were conducted to find significant differences between female and male patients who experienced diagnostic errors.

**Results:**

In total, 126 (7.4%) patients (of 1704) had been identified by case reviewers as having experienced at least 1 diagnostic error. Patients who had experienced diagnostic error were grouped by sex: 59 (7.1%) of the 830 women and 67 (7.7%) of the 874 men. Among the patients who experienced a diagnostic error, female patients were older (median 72, IQR 66-80 vs median 67, IQR 57-76; *P*=.02), had higher rates of being admitted through general or internal medicine (69.5% vs 47.8%; *P*=.01), lower rates of cardiovascular-related admitted diagnosis (11.9% vs 28.4%; *P*=.02), and lower rates of being admitted through neurology department (2.3% vs 13.4%; *P*=.04). The Ridge model achieved the highest area under the receiver operating characteristic curve (0.885), specificity (0.797), positive predictive value (PPV; 0.24), and *F*_1_-score (0.369) in classifying patients who were at higher risk of diagnostic errors among hospitalized patients.

**Conclusions:**

Our findings demonstrate that natural language processing can be a potential solution to more effectively identifying and selecting potential diagnostic error cases for review and therefore reducing the case review burden.

## Introduction

Diagnostic errors are an underappreciated cause of preventable mortality in hospitals, estimated to affect a quarter million hospital inpatients, and account for an estimated 40,000-80,000 deaths annually in the United States [1]. These errors pose a risk for severe patient harm [2,3], increase hospital length of stay [4], and made up 22% and accounted for US $5.7 billion of paid malpractice claims in hospitalized patients throughout a nearly 13-year period [5]. In their analysis of malpractice claims occurring in the US National Practitioner Database from 1999 to 2011, Gupta et al [5] found that diagnosis-related paid claims were most likely to be associated with death and cost (following surgery); among diagnosis-related paid claims, failure to diagnose was the most common subtype and was more likely than other types to be associated with mortality. Several factors have been proposed as contributors to inpatient diagnostic errors including time constraints related to the concurrent care of multiple patients, unpredictable workflows, distractions, and competing priorities for trainees. From their systematic review and meta-analysis, Gunderson et al [2] estimate that 250,000 diagnostic adverse events occur annually among hospitalized patients in the United States, and this is likely an underestimation of the problem due to several challenges in diagnostic error measurement [6].

Challenges in identifying and measuring diagnostic errors occur due to the evolving and iterative nature of the diagnostic process, making it difficult to determine when, if at all, a correct or more specific diagnosis could have been established by clinicians to start the appropriate treatment [6]. Since its landmark report, Improving Diagnosis in Health Care*,* the National Academies of Science, Engineering, and Medicine (NASEM) has produced a common understanding of diagnostic error that includes accuracy, timeliness, and communication of the explanation to the patient or patient’s family member [3]. Diagnostic errors often involve missed opportunities related to various aspects of the diagnostic process [7-9] and diagnostic adverse events resulting in harm [10]. However, many hospitals currently do not capture or include surveillance for diagnostic errors, despite having robust systems in place to report and analyze patient safety issues [6,11,12].

A crucial first step to improving diagnosis in hospitals is the creation of programs to identify, analyze, and learn from diagnostic errors. Ongoing efforts by the Agency for Health Care Research and Quality have supported pragmatic measurement approaches for health organizations to build a diagnostic safety program and identify and learn from diagnostic errors such as those described in the Measure Dx resource [9]. One proposed and promising solution for hospitals to improve diagnostic surveillance is to build on existing efforts to collect patient safety data, root cause analyses, or other forms of case reviews for quality improvement purposes. Cases that have already been reviewed or investigated in the organization for general patient safety and quality purposes may be able to inform or be rereviewed for information and learning opportunities specific to diagnostic safety. Widely used case-based learning methodologies in particular, such as the “Learning From Every Death” initiative developed at Mayo Clinic [13] used both nationally and worldwide, offer an excellent opportunity for hospitals to augment their existing quality and safety efforts and support diagnostic safety.

Clinical notes in electronic health records (EHRs) written by health providers in free-text format are rich sources of a patient’s diagnoses and care trajectory through hospitalization time. Approaches to processing free text, such as through natural language processing (NLP) and machine learning (ML), have demonstrated significant opportunities to improve quality and safety within health care organizations in diverse applications [14-16] such as cancer research [17,18] and infection prediction [19] to sleep issues [20] and neurological outcome prediction [21]. Besides its use in the diagnostic process, ML models proved to have added benefits when used in diagnostic error identification [22,23]. However, despite significant progress and evidence about the use of these ML and NLP approaches to improve patient safety, the use of ML and NLP approaches to diagnostic safety and surveillance has largely remained untapped. A 2022 study demonstrates how an academic medical center’s implementation of an NLP-based algorithm to flag safety event reports for manual review enabled early detection of emerging diagnostic risks from large volumes of safety reports, and was among the first to apply an NLP approach to safety event reports to facilitate identification of COVID-19 related diagnostic errors [24]. Meanwhile, progress in the use of data mining approaches to develop electronic trigger tools offers promising methods to detect potential diagnostic events, promote organizational learning, and support the monitoring of data prospectively to identify patients at high risk for future adverse events [25]. To our knowledge, however, NLP has not yet been applied to case review data to facilitate the identification of diagnostic errors and understand its features and sources.

While free-text formatted clinical notes provide unique opportunities to incorporate ML models, the lack of reliable labels to represent diagnostic errors often limits the use of clinical notes for diagnostic safety surveillance efforts. The opportunity to train ML and NLP algorithms to identify diagnostic errors and opportunities depends on the collation of EHR data with existing efforts to identify diagnostic errors such as through case review findings from the Safety Learning System (SLS). To further explore the potential for this approach to be used to improve diagnostic safety surveillance, a rigorous evaluation of the feasibility and potential of using EHR and existing case review data is needed.

We hypothesized that ML and NLP methods can be applied to train models based on available case review data to examine content potentially related to diagnostic errors within EHR clinical notes. These approaches automatically identify features or information from free text using controlled vocabularies, rule sets, reference dictionaries, or lexicons.

## Methods

### Data Sets and Case Review Approach

We analyzed SLS data from 1 large health system comprised of 10 hospitals in the mid-Atlantic region of the United States. The SLS is one example of a holistic case review methodology delivered by health care organizations in the United States and globally. Established in 2015, the SLS builds upon the Mayo Clinic Mortality Review System of Huddleston et al [13] to review and analyze EHR data from patient mortality cases to find safety issues that could be found and mitigated. This approach was designed to enhance current quality improvement projects done within health organizations, providing a perspective and strategy based on the Safety II lens and rooted in the belief that every death provides an opportunity to improve care. With a Safety II lens, participating organizations use a holistic case review methodology designed to identify vulnerabilities in systems and processes of care delivery. Reviewers identify and translate these into different categories and labels to (1) define and quantify types of process of care and system failures contributing to adverse outcomes (errors) and (2) identify the components of the process of care and system failures that when fixed will improve performance (opportunities for improvement [OFIs]).

To ensure a sufficient cross-sampling of patients across different specialties and areas, patients are selected for case reviews at this health system based on their primary provider service line category (eg, medicine, surgery, etc) and hospital length of stay; patients in primary and ambulatory care settings are not included for case review selection. The case review process occurs according to the standardized SLS methodology and recommendations [13,26], and between at least 1 physician and 1 nurse within the health system who have both received training in the SLS approach. The case review outcome and identification of OFIs, including diagnostic OFIs, relies on the reviewer’s consensus of any findings and through multiple multidisciplinary and multispecialty meetings that may involve a committee Chair member, clinical department leader, or escalation to other leadership.

### Sample

We obtained SLS data from February 2016 to September 2021; data in later years were available but not included because of key changes to the case selection process made during and in response to the COVID-19 pandemic. All hospitalized adult patients older than 18 years were included for analysis, regardless of their hospitalization outcome (eg, mortality or discharge location). Pediatric and neonatal patients were excluded.

### Ethical Considerations

The original data collection and study protocol was approved by the institutional review board (00001245) at MedStar Health Research Institute on August 26, 2019.

### Variables

#### Data Extraction

Medical record number, encounter number, length of stay, age, date of birth, sex, diagnosis at the time of admission (ie, *ICD-10* [*International Statistical Classification of Diseases, Tenth Revision*] diagnosis codes), mortality, OFI categories (eg, delayed or missed diagnosis and diagnostic opportunities), number of identified OFIs and diagnosis issues (eg, the accuracy of diagnosis and confirmation or fixation bias) were the features and patient identifiers which were extracted from SLS data [13,26].

Because chart reviews generally occur at a single point in time within the patient’s care trajectory, they often do not contain information or details of the patient’s full hospital course. However, clinical notes written by health care providers are rich sources of patient’s health status throughout their hospitalization period [27-29]. Therefore, to supplement these chart review data, we additionally extracted and included all clinical notes from the EHR for patients who could be matched by patient identifiers (eg, encounter number and date of birth).

#### Coding Diagnostic Errors

Case reviewers can select any number of labels to describe a diagnosis issue or an OFI identified and agreed upon by consensus. For this study, diagnostic errors were defined by the available features from chart review pertaining to diagnosis and impacting the timeliness, accuracy, or communication of a diagnosis. Our definition of diagnostic errors was limited to the categories identified during chart reviews and recorded within the SLS data set; therefore, our diagnostic error definition does not include all aspects of the definition developed by the NASEM report [3]. Table 1 describes the SLS categories and values that were labeled as diagnostic errors and used to train our classification models. Patients were coded as having experienced a diagnostic error if one or more of the conditions listed in Table 1 were identified in their SLS case review.

**Table 1:** Indicators of diagnostic error in Safety Learning System data.

Feature from chart reviews	Value to indicate diagnostic error
OFI^a^ category	Delayed or missed diagnosis
OFI category	Diagnostic opportunities
Diagnosis issues	accuracy of diagnosis
Diagnosis issues	Accuracy of interpretation of laboratory or test results
Diagnosis issues	Squirrel (red herring lab or test results)
Diagnosis issues	Confirmation or fixation bias
Diagnosis issues	Appropriateness of chosen tests or equipment given the patient’s differential diagnosis

^a^OFI: opportunity for improvement.

#### NLP Approach

We used an NLP approach on critical incident reporting system data to explore the features and risk of diagnostic error among hospitalized patients.

#### Features From Free-Text Data

Descriptive statistical analyses were performed to identify any differences among age, length of stay, and mortality between the female and male patients who had experienced diagnostic errors.

All EHR clinical notes were transformed to lowercase. Extra white spaces, numbers, punctuations, and stop words were removed and words were stemmed. The term frequency-inverse document frequency (TF-IDF) matrix was calculated for each clinical note using the bag-of-words from the preprocessed EHR clinical notes [30]. TF-IDF is a statistical measure that evaluates how relevant a word is to a document in a collection of documents and is a popular method to translate free text to numerical features in training ML models. The TF-IDF of a word in a document is calculated by multiplying 2 metrics: the number of times a word appeared in a document and the inverse document frequency of the word across a set of documents. TF-IDF is computationally efficient and easy to interpret. We excluded the most frequent words that had appeared in more than 95% of the EHR clinical notes, as these frequent words do not provide information to help with the classification. Moreover, we excluded the rare words that appeared in less than 5% of the EHR clinical notes [31].

In a TF-IDF matrix, the number of rows corresponds to the unique patients, and the number of columns represents the unique words found in EHR clinical notes. There are numerous unique words used in EHR clinical notes; therefore, the TF-IDF approach provides a high-dimensional input matrix for the classification task. The high-dimensional input matrix can lead to training inaccurate classifiers. To overcome that issue, we used the chi-square statistical test to select the most relevant words to identify diagnostic errors; therefore, if *P* values associated with a word (also called a feature) are less than .05, that word is selected and included in the feature matrix to train ML classification models.

#### Classification Models

In lieu of an existing model with the same objective in the literature, a simple logistic regression model was trained as the baseline classifier to identify patients within SLS data who were at higher risk of diagnostic error. Moreover, 3 forms of logistic regression models with regularization functions were trained on this data to compare classification performances and identify the best-performing model [32]: Least Absolute Shrinkage and Selection Operator (LASSO), Ridge, and Elastic Net.

- LASSO: for a more accurate prediction, LASSO regularization is used with a logistic regression model. The LASSO procedure encourages simple, sparse models which has fewer parameters in a way that the estimated coefficient of features with less effect will be set to zero. This characteristic makes LASSO well-suited for models showing high levels of multicollinearity or variable selection and parameter elimination is needed. LASSO is also called L1 regularization.
- Ridge: also called L2 regularization, Ridge is a regularization method used for models suffering from multicollinearity or high-dimensional feature space. Ridge regularization keeps all the features regardless of their effect on the model. However, it pushes the estimated coefficient of features with less effect toward zero to minimize their effect on the classification outcome. This characteristic of Ridge makes it well-suited when most features impact the outcome variable.
- Elastic Net: a logistic regression model with Elastic Net regularization is a weighted combination of LASSO (L1) and Ridge (L2) regularizations [33]. Elastic Net can remove the effect of the insignificant features by setting their estimated coefficient to zero and lower the effect of the less significant features by pushing their estimated coefficient toward zero while adding more weights to the more important features. From implementation and interpretation aspects, the Elastic Net model is simple to use. Such characteristics make this model an accepted baseline in ML-based studies [34].

The hyperparameters of the 3 classification models were optimized through cross-validation. All the analyses were conducted using Python 3 (Python Software Foundation).

#### Classification Performance Metrics

**Figure 1:**
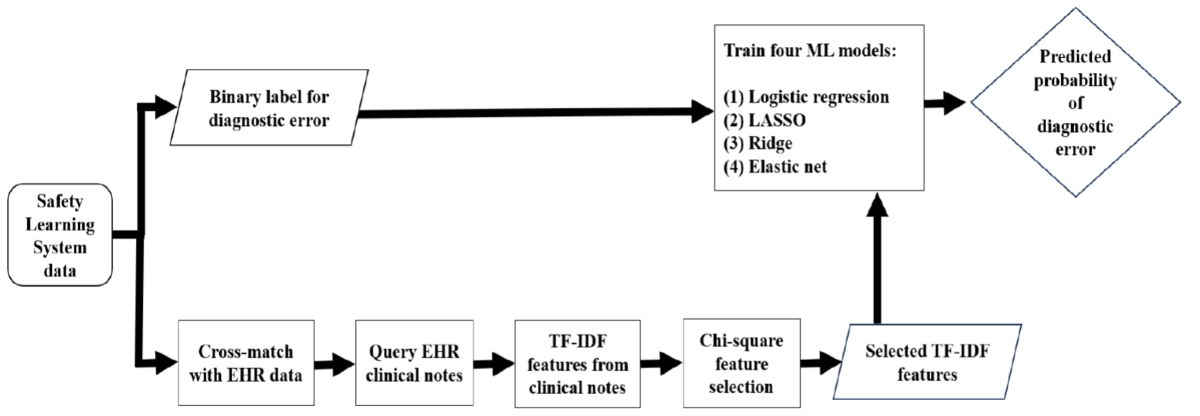
A summary of the methods used in this study. EHR: electronic health record; LASSO: Least Absolute Shrinkage and Selection Operator; TF-IDF: term frequency-inverse document frequency.

We calculated 7 common performance metrics reported for binary classifiers to compare the performance of the 4 classification models: area under receiver operating characteristics curve (AUROC), sensitivity or recall or true positive rate, specificity or true negative rate, positive predictive value (PPV) or precision, negative predictive value (NPV), *F*_1_-score, and area under precision-recall curve (AUPRC). The 7 metrics take values between 0 and 1. Values closer to 1 indicate a well-performing classifier. Multimedia Appendix 1 presents the definition of the performance metrics used in this study. Figure 1 presents the summary of the methods used in this analysis.

## Results

### Descriptive Summary

In total, there were 2184 unique patient records within SLS data from February 2016 to September 2021. EHR clinical notes were cross-matched, extracted, and included in analyses for 1704 (78%) of these SLS patient records. Of those patients with cross-matched EHR data, 126 (7.4%) patients had been identified by case reviewers as having experienced at least 1 diagnostic error. A total number of 20,848 EHR clinical notes associated with the 1704 unique patients were used in this study.

Patients who had experienced diagnostic errors were grouped by sex: 59 (7.1%) of the 830 women and 67 (7.7%) of the 874 men in the larger cross-matched sample had been found to have a diagnostic error. Table 2 presents the descriptive statistics between female and male patient groups. We applied the Wilcoxon rank sum test for numerical features (ie, age and length of stay), and the chi-square test for mortality rate, admission diagnosis, and admission department or specialty. Patients in the female group were older than the male group by a median of 72 (IQR 66-80) versus a median of 67 (IQR 57-76; *P*=.02). Compared to the male group, female patients who experienced diagnostic error had higher rates of being admitted through general or internal medicine (69.5% vs 47.8%; *P*=.01), lower rates of cardiovascular-related admitted diagnosis (11.9% vs 28.4%; *P*=.02), and lower rates of being admitted through neurology department (2.3% vs 13.4%; *P*=.04). We observed no differences between groups in mortality rates and length of stay.

**Table 2:** Descriptive statistics of patients.

			Patients who experienced diagnostic error					All patients		
			Female group (n=59)		Male group (n=67)		Female group (n=830)			Male group (n=874)
Age (in years), median (IQR)			72 (66-80)		67 (57-76)		72 (62-83)			69 (59-79)
**Race, n (%)**										
	African American	38 (64)		42 (62)		429 (51.7)			429 (51.7)	
	Asian	0 (0)		0 (0)		12 (1.4)			12 (1.4)	
	Multiple	0 (0)		0 (0)		2 (0.2)			2 (0.2)	
	Not recorded	4 (6)		2 (2.9)		30 (3.6)			30 (3.6)	
	White	11 (18)		21 (31.3)		310 (37.3)			310 (37.3)	
	Other	6 (10)		2 (2.9)		47 (5.7)			47 (5.7)	
Length of stay in days, median (IQR)			4 (6-10)		4 (8-14)		7 (4-12)			8 (4-12)
**Mortality, n (%)**										
	Count	25 (42)		29 (43)		456 (54.9)			459 (52.5)	
**Admitting department or specialty, n (%)**										
	General or internal medicine or hospitalist	41 (69)		32 (47)		427 (51.4)			389 (44.5)	
	Cardiology	5 (8)		12 (17)		99 (11.9)			131 (14.9)	
	Critical care	6 (10)		6 (8)		117 (14.1)			142 (16.2)	
	Neurology	2 (3)		9 (13)		75 (9)			90 (10.3)	
	Pulmonary	1 (1)		1 (1)		22 (2.6)			31 (3.5)	
	Other	4 (6)		7 (10)		90 (10.8)			91 (10.4)	
**Admitting diagnosis, n (%)**										
	Cardiovascular	7 (11)		19 (28)		154 (18.6)			167 (19.1)	
	Respiratory	7 (11)		5 (7)		88 (10.6)			69 (7.9)	
	Sepsis	7 (11)		4 (5)		65 (7.8)			63 (7.2)	
	Altered mental status	1 (1)		2 (2)		36 (4.3)			28 (3.2)	
	Diabetes	1 (1)		1 (1)		6 (0.7)			3 (0.3)	
	Other	23 (38)		21 (31)		244 (29.4)			270 (30.9)	
**Admitting unit type, n (%)**										
	General care	54 (91)		60 (89)		144 (17.3)			179 (20.5)	
	Critical care	5 (8.5)		7 (10)		686 (82.7)			695 (79.5)	
**OFI^a^ categories, n (%)**										
	Delayed or missed diagnosis	43 (72)		46 (68)		43 (5.2)			46 (5.3)	
	Diagnostic opportunities	15 (25)		16 (23)		15 (1.8)			16 (1.8)	
	Accuracy of diagnosis	1 (1)		4 (6)		1 (0.1)			4 (0.5)	
	Accuracy of interpretation of laboratory or test results	0 (0)		0 (0)		0 (0)			0 (0)	
	Squirrel (red herring lab or test results)	0 (0)		1 (1)		0 (0)			1 (0.1)	
	Confirmation or fixation bias	0 (0)		0 (0)		0 (0)			0 (0)	
	Appropriateness of chosen tests or equipment given patient’s differential diagnosis	1 (1)		0 (0)		1 (0.1)			0 (0)	
**OFI unit type, n (%)**										
	Critical care	15 (25)		22 (32)		273 (32.9)			318 (36.4)	
	Emergency department	17 (28)		18 (26)		81 (9.8)			76 (8.7)	
	General care	27 (45)		27 (40)		290 (34.9)			285 (32.6)	

^a^OFI: opportunity for improvement.

### Classification Models’ Performance

Clinical notes were preprocessed for TF-IDF feature calculation. The bag-of-words included 2227 words, and each word was considered a feature (see Table S1 in Multimedia Appendix 2 for the top 100 words). We found that abscess, ascend, abnormality, scant, pair, and prefer were the top 5 features with the highest positive estimated coefficient (0.42 to 0.28); post, select, gave, muscl, hours, and unrespons were the top 5 features with the highest negative coefficients (–0.35 to –0.26). After applying the chi-square test, 250 features with a *P* value less than .05 were selected for the modeling process. All 4 ML classifiers were trained using the 250 selected features.

Table 3 presents the performances of the simple logistic regression and 3 regularized logistic regression models (LASSO, Ridge, and Elastic Net). The Ridge model achieved the highest AUROC (0.885), specificity (0.797), PPV (0.24), NPV (0.981), and *F*_1_-score (0.369) in classifying patients who were at higher risk of diagnostic errors among hospitalized patients in SLS system. The simple logistic regression model obtained the highest AUPRC (0.537). The simple logistic regression model classified all patients as the ones with diagnostic errors; therefore, it achieved a sensitivity of 1, and specificity and NPV of 0.

**Figure 2:**
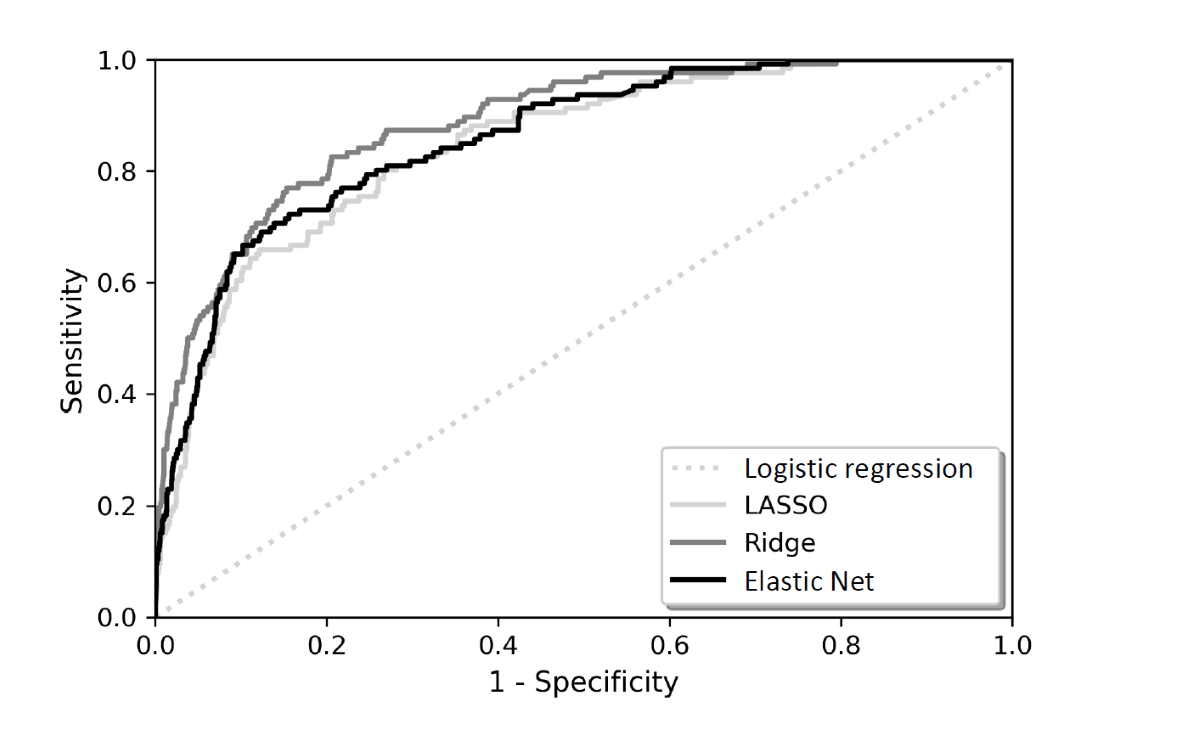
Receiver operating characteristics curves. LASSO: Least Absolute Shrinkage and Selection Operator.

**Figure 3:**
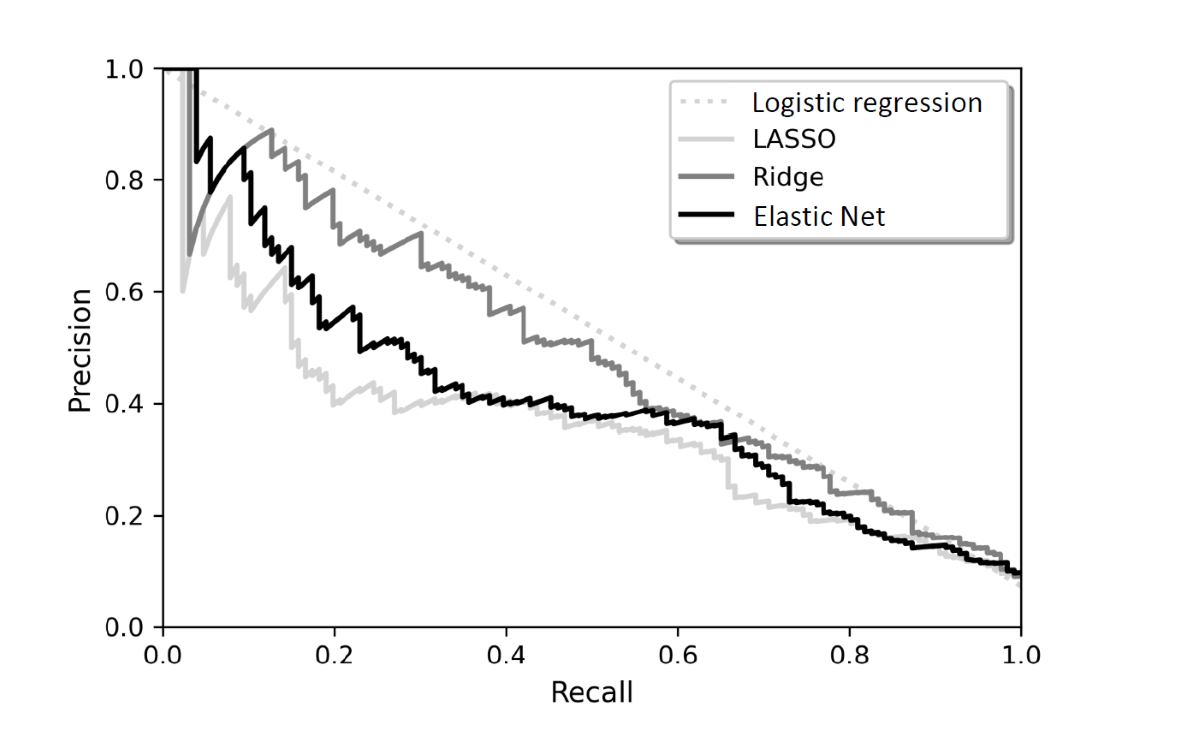
Precision recall curves. LASSO: Least Absolute Shrinkage and Selection Operator.

Figures 2 and 3 present the receiver operating characteristics curves and precision-recall curves for the 4 classifiers in this study. Models that give ROC curves closer to the top-left corner indicate a better performance. The AUROC values represent the probability that a patient who experienced a diagnostic error, chosen at random, is ranked higher by the Ridge model than a randomly chosen patient who did not experience a diagnostic error. The higher value of AUPRC indicates that the Ridge model can identify patients who experienced diagnostic errors more precisely with fewer false positives compared to LASSO and Elastic Net models.

**Table 3:** Performance metrics. The performance metrics of 4 machine learning algorithms in classifying patients who are at higher risk of diagnostic errors among hospitalized patients. The numbers represent the average of the 5-fold cross-validation approach.

	Simple logistic regression	LASSO^a^	Ridge	Elastic Net
AUROC^b^	0.5	0.846	0.885	0.859
Sensitivity	1.0	0.802	0.802	0.802
Specificity	0	0.733	0.797	0.742
Positive predictive value	0.074	0.193	0.24	0.199
Negative predictive value	0	0.979	0.981	0.979
*F*_1_-score	0.138	0.312	0.369	0.319
AUPRC^c^	0.537	0.361	0.491	0.411

^a^LASSO: Least Absolute Shrinkage and Selection Operator.

^b^AUROC: area under receiver operating characteristics curve.

^c^AUPRC: area under precision-recall curve.

## Discussion

### Principal Findings

Our contribution is 2-fold; first, we integrated 2 data sources that are currently used by and available to many organizations across the United States, SLS and EHR data, to explore the use of ML and NLP algorithms to help identify diagnostic errors among hospitalized patients. Although case review methodologies offer rich insights into systems errors and OFIs, the predefined pull-down menus and structured data labels typically do not capture all the necessary clinical and contextual details that are considered by reviewers. Therefore, a large portion of these case review data are stored in free-text narratives that typically record key information and judgments decided upon by the multidisciplinary reviewers. However, given persistent issues of staff shortage and lack of time in health care settings, it is becoming increasingly important to lower the burden of systematic EHR data reviews for health care providers while maintaining the review systems in place. Second, any developed ML and NLP approaches can potentially be incorporated to generate a diagnostic error risk score for each patient. The predicted risk score can be used in identifying and prioritizing patients for focused chart reviews, thus lowering the burden of systematic EHR data reviews for health care providers while maintaining the review systems in place.

To our knowledge, this study is the first attempt to apply and test several different ML classification models to identify diagnostic errors within routinely collected organizational case review data. Despite a substantial body of literature about the prevalence of diagnostic errors in hospital settings, current efforts to identify diagnostic errors generally rely on reviews of patient cases and data by clinical or quality teams that often are resource-intensive. ML classification models and NLP techniques offer an opportunity to generate diagnostic error risk scores to sort through large data sets and identify signals of potential diagnostic errors that can be flagged for further review. However, these classification models require a high number of observations (and identified diagnostic errors) to perform well, which might not be feasible for health organizations that are just beginning to identify diagnostic errors or may have limited personnel and efforts to perform high numbers of case reviews. In this study, we accessed nearly 2000 patient records (and of those, only 126 cases of diagnostic errors), which is considered to be a limited data sample size in the field of ML. However, techniques, such as feature selection and n-fold cross-validations, can potentially be approaches to address small sample size challenges [35].

Using the results of the simple logistic regression model as the baseline performance, we found that 3 regularization functions, namely LASSO, Ridge, and Elastic Net, boosted the performance of the baseline model. The Ridge model outperformed the rest of the models in terms of multiple performance metrics: AUROC of 0.885, specificity of 0.797, PPV of 0.24, NPV of 0.981, and *F*_1_-score of 0.369. The Ridge algorithm tries to keep all features in the model even the features with a slight effect on the classification outcome. Since the patterns pointing at a diagnostic error were subtle in the clinical notes, even a small effect of a feature on the model’s classification outcome could be important for the classification model to learn. On the other hand, the LASSO algorithm rigorously removes features that have a small effect on the classification outcome. The Elastic Net model is a weighted combination of LASSO and Ridge. The performance results presented in Table 3 show that the values achieved by the Elastic Net model lie between those of the LASSO and Ridge models.

### Insights From Diagnostic Errors Within Free-Text Clinical Notes

We did not find the free text formatted clinical notes in the EHR to reflect any sort of direct language around diagnostic errors. Our analysis identified no use of the terms misdiagnosis, missed diagnosis, or diagnostic error within clinical notes, finding instead more subtle signals pointing at diagnostic errors such as “there may be a chance of misreading the test,” or “insufficient data to make a diagnosis.” Our findings demonstrate that NLP algorithms can be used to identify such patterns and find the associations between diagnostic errors and the subtle signals in the clinical notes. A natural extension of this work can focus on using other feature extraction methods, such as Bidirectional Encoder Representations from Transformers contextualized word embeddings, and explore the use of the pretrained language models for this objective.

We found that the presence of terms, such as abscess, abnormality, “cp” (chest pain)*,* and dialysis in a patient’s EHR clinical note were associated with reviewer-identified diagnostic errors (Multimedia Appendix 2). Misinterpretation of chest pain, specifically among female patients, has the potential to cause a cardiovascular-related diagnosis error [36]. Patients with chronic kidney disease are at higher risk of cardiovascular complications [37]. Missing such risk for a patient who is on dialysis, adds to the risk of diagnostic error.

### Clinical and System Implications Around Diagnostic Inequity

Diagnostic inequity is defined as “the presence of preventable unwarranted variations in diagnostic process among population groups that are socially, economically, demographically, or geographically disadvantaged” [38]. Despite persistent and well-documented disparities in health care access and outcomes across different population groups, few studies have examined the association between diagnostic errors and health care disparities [39]. Recent evidence supports the notion that variation in diagnostic error rates across demographic groups may exist, particularly across sex. A systematic review of diagnostic errors in the emergency department, for example, found that female sex and non-White race were often associated with increased risk for diagnostic errors across several clinical conditions in emergency settings [40]. In cardiovascular medicine, a national cohort study of acute myocardial infarctions found that women were nearly twice as likely as men to receive the wrong initial diagnosis following signs of a heart attack [41]. Despite efforts to understand and reduce disparities in diagnosis and treatment, women not only continue to be understudied, underdiagnosed, and undertreated in cardiovascular medicine [42] but also may experience longer lengths of time to diagnosis than men in most patterns of disease diagnosis [43].

The analysis of case review data and other system-based data (eg, patient safety events or incident reporting) by subsets offer an opportunity to identify events in vulnerable patient populations and help sensitize clinicians to potential biases within the diagnostic process. To explore sex differences in diagnostic errors within our case review data, we statistically compared demographic and clinical differences between female and male patients who had been identified in case reviews as having experienced diagnostic error or errors. We found that of those patients who had experienced diagnostic error or errors, the female group of patients were older, had higher rates of being admitted through general or internal medicine or hospitalist (vs specialty) departments, and had lower rates of having a cardiovascular diagnosis on admission. These preliminary results of this study revealed unexpected differences between male and female diagnostic error groups, offering novel insights that warrant further investigation to fully understand the mechanisms underlying these relationships and their implications for clinical decision-making and practice. Future uses of NLP can potentially support clinical and system-based approaches to capture and increase the evidence around structural biases or disparities in diagnoses. Individual cases from these types of data sources could be used as example narratives to engage clinicians and improve clinician learning, contributing to the development of tailored clinician and systemic interventions that can improve quality and equity throughout the diagnostic process.

### Limitations

This study has several limitations. Our definition of diagnostic errors was limited to the categories and labels used within the SLS data set, reviewer interpretations of cases (subject to reviewer bias), and does not include all aspects of the definition developed by the NASEM report [3]. Despite several continued differences in definitions of diagnostic error in the peer-reviewed literature [8], we recommend that quality and safety teams within health systems use the NASEM definition for diagnostic error—including errors in communicating the diagnosis to the patient—to develop any definitions, categories, or labels used in their case review and surveillance initiatives. Although a time-consuming task, future studies could consider EHR data chart reviews to have the ground truth for the diagnostic error cases and add to the accuracy of the data set used for training the ML classifiers. Additionally, due to staffing challenges and shifting organizational priorities, case review selection varies by hospital and has changed over time, resulting in a relatively small sample size and also introducing the potential for bias. Our data came from a single health system and may reflect the specific language, culture, and practices occurring within the system and therefore may not be similar to that of other health systems. To enhance the external validity and generalizability of results, future efforts and research studies should consider the random selection of cases to evaluate both diagnostic and general quality issues within the organization; studies with larger sample sizes can build on our preliminary findings and test differences between clinical subgroups. Finally, our classification models were developed and evaluated based on a retrospective cohort from EHR; therefore, the performance may deteriorate when the method is applied to real-time data. Further work or future studies should be conducted to prospectively validate the models.

### Conclusions

We performed an NLP approach and compared 4 techniques to classify patients who were at a higher risk of experiencing diagnostic error during hospitalization. Our findings demonstrate that NLP can be a potential solution to more effectively identifying and selecting potential diagnostic error cases for review, and therefore, reducing the case review burden.

## Appendix

Multimedia Appendix 1 Binary classification performance metrics.

Multimedia Appendix 2 The Estimated Coefficient from the Ridge Model.

## Abbreviations

AUPRC: area under precision-recall curve
AUROC: area under receiver operating characteristic curve
EHR: electronic health record
ICD-10: International Statistical Classification of Diseases, Tenth Revision
LASSO: Least Absolute Shrinkage and Selection Operator
ML: machine learning
NASEM: National Academies of Science, Engineering, and Medicine
NLP: natural language processing
NPV: negative predictive value
OFI: opportunity for improvement
PPV: positive predictive value
SLS: Safety Learning System
TF-IDF: term frequency-inverse document frequency
