# Supplementary material for: Evaluation of a Natural Language Processing Approach to Identify Diagnostic Errors and Analysis of Safety Learning System Case Review Data: Retrospective Cohort Study
Source: J Med Internet Res. 2024 Aug 26;26:e50935. doi: 10.2196/50935 (PMC11384169; doi:10.2196/50935)
Supplement: Multimedia Appendix 1 [file jmir_v26i1e50935_app1.docx]

| **Performance Metrics** | **Definition** |
| --- | --- |
| Area Under Receiver Operating Characteristics Curve (AUROC) | AUROC of a classifier is the probability that the model ranks a patient with diagnostic error more highly than a patient with timely and correct diagnosis. |
| Sensitivity or True Positive Rate or Recall | The sensitivity of a classifier is its ability to designate a patient who had experienced diagnostic error as positive. |
| Specificity or True Negative Rate | The specificity of a classifier is its ability to designate a patient who did not experience diagnostic error as negative. |
| Positive Predictive Value (PPV) or Precision | PPV of a classifier is the probability that a patient who was classified to experience a diagnostic error actually had experienced a diagnostic error. |
| Negative Predictive Value (NPV) | NPV of a classifier is the probability that a patient who was classified to experience an accurate and timely diagnosis, actually had not experienced a diagnostic error. |
| F-1 Score | F-1 score is the harmonic mean of sensitivity and PPV. F-1 score is a measure of a classifier’s accuracy in identifying patients who experienced diagnostic error. |
| Area Under Precision-Recall Curve (AUPRC) | The precision-recall curve shows the tradeoff between precision (or PPV) and recall (or sensitivity). A high AUPRC represents both high recall and high precision. |
