# Supplementary material for: Evaluation of a Natural Language Processing Approach to Identify Diagnostic Errors and Analysis of Safety Learning System Case Review Data: Retrospective Cohort Study
Source: J Med Internet Res. 2024 Aug 26;26:e50935. doi: 10.2196/50935 (PMC11384169; doi:10.2196/50935)
Supplement: Multimedia Appendix 2 [file jmir_v26i1e50935_app2.docx]

The bag-of-words from EHR clinical notes included 2,227 words. Chi-square feature selection was applied and 250 words (also referred to as features) were selected. Table SB-1 presents the first 100 selected words along with their associated estimated coefficient from the Ridge model. Having features with negative estimated coefficients in a patient’s clinical notes decreases the probability of the patient experiencing diagnostic error.

Table S1. The words which were utilized in training the classification models. Words were stemmed and sorted based on absolute values of estimated coefficients.

| **Feature** | **Positive Estimated Coefficient** | **Feature** | **Negative Estimated Coefficient** |
| --- | --- | --- | --- |
| abscess | 0.42158 | post | -0.35521 |
| ascend | 0.40654 | select | -0.34104 |
| abnormality | 0.3482 | gave | -0.2884 |
| scant | 0.28128 | muscl | -0.2662 |
| pair | 0.27772 | hours | -0.26325 |
| prefer | 0.27598 | unrespons | -0.24526 |
| cp | 0.26657 | receiv | -0.2438 |
| dialysi | 0.26483 | describ | -0.24134 |
| subcutan | 0.26464 | fentanyl | -0.24118 |
| activ | 0.2641 | velocity | -0.22382 |
| otherwise | 0.26305 | band | -0.22192 |
| administered | 0.25899 | dnrdni | -0.22153 |
| info | 0.25599 | her | -0.21753 |
| caliber | 0.25008 | imaging | -0.2121 |
| performed | 0.23921 | speak | -0.21179 |
| enrol | 0.23723 | valdiviezoschlomp | -0.20179 |
| stone | 0.23699 | intraventricular | -0.19742 |
| nonobstruct | 0.2358 | alcohol | -0.19589 |
| emerg | 0.23397 | pressure | -0.19242 |
| multifoc | 0.23054 | gallbladd | -0.18914 |
| needs | 0.22991 | start | -0.18706 |
| type | 0.22626 | bag | -0.18353 |
| mm | 0.22528 | record | -0.18237 |
| maryland | 0.22447 | ecg | -0.17347 |
| abd | 0.22363 | hous | -0.16994 |
| improved | 0.22004 | whcne | -0.16972 |
| midline | 0.21752 | appl | -0.16959 |
| written | 0.2163 | comment | -0.16926 |
| clinic | 0.20588 | intens | -0.16624 |
| stretcher | 0.20111 | wit | -0.1656 |
| entri | 0.19564 | pull | -0.16053 |
| gland | 0.1925 | transfer | -0.15819 |
| from | 0.191 | signatur | -0.15727 |
| consist | 0.18839 | expir | -0.15568 |
| hemostasi | 0.18346 | echo | -0.155 |
| adren | 0.18292 | encephalopathy | -0.14991 |
| apart | 0.18274 | barrier | -0.14902 |
| departm | 0.18134 | recommendations | -0.14783 |
| scatter | 0.17858 | npe | -0.14753 |
| ultrasound | 0.17758 | famili | -0.14662 |
| unremark | 0.17111 | atherosclerot | -0.14575 |
| fluid | 0.1673 | larg | -0.14505 |
| product | 0.16657 | bilirubin | -0.14409 |
| hydronephrosis | 0.16567 | overli | -0.1439 |
| manual | 0.16417 |  |  |
| bowel | 0.16245 |  |  |
| sonograph | 0.1604 |  |  |
| pneumonia | 0.16003 |  |  |
| diameter | 0.15991 |  |  |
| fluoroscopi | 0.15784 |  |  |
| abdomen | 0.15169 |  |  |
| presenc | 0.1467 |  |  |
| pelvic | 0.14482 |  |  |
| th | 0.14289 |  |  |
| renal | 0.14238 |  |  |
| deliv | 0.1417 |  |  |
